# Analysis of Intraviral Protein-Protein Interactions of the SARS Coronavirus ORFeome

**DOI:** 10.1371/journal.pone.0000459

**Published:** 2007-05-23

**Authors:** Albrecht von Brunn, Carola Teepe, Jeremy C. Simpson, Rainer Pepperkok, Caroline C. Friedel, Ralf Zimmer, Rhonda Roberts, Ralph Baric, Jürgen Haas

**Affiliations:** 1 Genzentrum, Max-von-Pettenkofer-Institut, Lehrstuhl Virologie, Ludwig-Maximilians-Universität (LMU), München, Germany; 2 European Molecular Biology Laboratory (EMBL) Heidelberg, Heidelberg, Germany; 3 Institut für Informatik, Ludwig-Maximilians-Universität (LMU), München, Germany; 4 University of North Carolina at Chapel Hill, Chapel Hill, North Carolina, United States of America; University of Hong Kong, China

## Abstract

The severe acute respiratory syndrome coronavirus (SARS-CoV) genome is predicted to encode 14 functional open reading frames, leading to the expression of up to 30 structural and non-structural protein products. The functions of a large number of viral ORFs are poorly understood or unknown. In order to gain more insight into functions and modes of action and interaction of the different proteins, we cloned the viral ORFeome and performed a genome-wide analysis for intraviral protein interactions and for intracellular localization. 900 pairwise interactions were tested by yeast-two-hybrid matrix analysis, and more than 65 positive non-redundant interactions, including six self interactions, were identified. About 38% of interactions were subsequently confirmed by CoIP in mammalian cells. Nsp2, nsp8 and ORF9b showed a wide range of interactions with other viral proteins. Nsp8 interacts with replicase proteins nsp2, nsp5, nsp6, nsp7, nsp8, nsp9, nsp12, nsp13 and nsp14, indicating a crucial role as a major player within the replication complex machinery. It was shown by others that nsp8 is essential for viral replication *in vitro*, whereas nsp2 is not. We show that also accessory protein ORF9b does not play a pivotal role for viral replication, as it can be deleted from the virus displaying normal plaque sizes and growth characteristics in Vero cells. However, it can be expected to be important for the virus-host interplay and for pathogenicity, due to its large number of interactions, by enhancing the global stability of the SARS proteome network, or play some unrealized role in regulating protein-protein interactions. The interactions identified provide valuable material for future studies.

## Introduction

The observation of atypical pneumonias in the Chinese province Guangdong in November 2002 led to the identification of the severe acute respiratory syndrome (SARS). Within a few months, the disease spread to a large number of countries and caused more than 8,000 cases and almost 800 deaths. The causative pathogen identified was shown to be a new human coronavirus designated the SARS-CoV [1]. Tight intervention strategies limited the further spread of the pathogen. Sequence analysis of the first isolates of the newly identified SARS-CoV revealed characteristic features typical of the known three coronavirus groups [2]–[4]. According to several phylogenetic analyses the virus is grouped either a novel group IV or an early split-off of group II coronaviruses [5], [6].

The genome of the SARS-CoV consists of a positive-stranded RNA of approximately 29,700 nt in length. The replicase genes span the first two-thirds of the genome containing the two overlapping ORF1a and ORF1b, which are connected by a ribosomal frameshift. The two polyproteins expressed are predicted to encode and to be cleaved by a papain-like proteinase 2 (PL-Pro = part of nsp3) and a 3C-like proteinase (3CL-Pro = nsp5) to 16 mature replicase proteins [1], [6]. They are well conserved between SARS-CoV and other coronaviruses. It is suggested that they are required for the synthesis of the full-length genome and subgenomic RNA synthesis as well as for virus replication [6], [7]. Functions of the processed proteins include a single-stranded RNA-binding protein (nsp9) an RNA-dependendent RNA polymerase (RdRp = nsp12) as well as a non-canonical RdRp (nsp8) synthesizing short primers for nsp12, a superfamily 1-like helicase (HEL1 = nsp13), and a uridylate-specific endoribonuclease (NendoU = nsp15) [7]–[15]. Nsp3, nsp14, nsp16 are thought to have ADP-ribose 1′-phosphatase, 3′->5′ exonuclease and 2′-O-ribose methyltransferase activities, respectively [6]. But many of the functions of the nsps are still unknown. At their 5′- terminus the subgenomic mRNAs share a common leader sequence encoded at the 5′- end of the genome, which is joined to the respective gene sequences at specific transcription regulatory sequences. Their common 3′- ends extend to the end of the genome.

The last third of the genome encodes the S, E, M and N structural genes with the group-specific genes interspaced among them. Former are encoded by mRNAs 2, 4, 5, and 9, latter by transcripts 3, 6, 7, 8, and 9, respectively. These genes, ORF3a/b, ORF6, ORF7a/b, ORF8a/b, and ORF9b are not found in other coronaviruses and their functions with respect to replication and pathogenesis are not well understood. There is evidence that some of the accessory ORFs can be deleted individually or in combination with almost no impact on *in vitro* growth, RNA synthesis, or on *in vivo* virus replication in a murine model [16]. Also, the nsp2 replicase protein of murine hepatitis virus (MHV) and SARS-CoV is dispensable for virus replication in cell culture. Its deletion results in attenuation of viral growth and RNA synthesis [17]. There are reports that a number of MHV and SARS-CoV replicase proteins colocalize and eventually interact in cytoplasmic membrane bound complexes, in which viral RNA synthesis occurs [18], [19]. Direct interactions of nsp7 and nsp8 in a hexadecameric supercomplex could be demonstrated by crystallography [20]. Interactions of the structural N and M proteins were demonstrated by a mammalian two-hybrid system [21].

For the elucidation of molecular mechanisms during the course of viral growth and propagation there is a need to systematically examine possible interactions of all viral proteins. We therefore cloned the SARS-CoV ORFeome by recombinatorial cloning (GATEWAY technology) and performed a genome-wide analysis for viral protein interactions by yeast-two-hybrid (Y2H) matrix screen.

## Results

### Generation of a SARS-CoV ORFeome

We have designed a set of nested PCR primers to amplify all viral non-structural, structural and accessory ORFs at the predicted protease cleavage sites or at the respective start and stop codons (Table 1). For cloning reasons, nsp3 was subdivided into a N-terminal (nsp3N, nt positions 2719–4431) and a C-terminal (nsp3C, nt positions 4885–8484) fragment containing the ADP-ribose-1”monophosphatase domain and the Papain-like proteinase, respectively. An accessory ORF14 described only by Marra et al. was also included [3]. Primers were designed such that they contained gene-specific sequences for the amplification of the respective ORFs. Overhanging sequences made them compatible to the Gateway® recombinatorial cloning system allowing the cloning into a so-called pDONR207 vector with the subsequent subloning into the destination vectors pGADT7-DEST (prey) and pGBKT7-DEST (bait).

**Table 1:** SARS-CoV Orfs used for construction of the viral orfeome

Protein	Predicted AA	No. AA	Cloned nt	Protein	No. AA	Cloned nt
Non-structural proteins:				Structural proteins:		
nsp1	M1-180G	180	265–804	S	1255	21492–25259
nsp2	A181-818G	638	805–2718	E	76	26117–26347
nsp3	A819-G2740	1922	2719–8484	M	221	26398–27063
nsp3N: (ADRP)	A819-1389E		2719–4431			
nsp3C: (PLP)	E1541-2740G		4885–8484			
nsp4	K2741-3240Q	500	8485–9984	N	422	28120–29388
nsp5: 3CLpro	S3241-3546Q	306	9985–10902			
nsp6	G3547-3836Q	290	10903–11772	**Accessory proteins:**		
nsp7	S3837-Q3919	83	11773–12021	3a	274	25268–26092
nsp8	A3920-Q4117	198	12022–12615	3b	154	25689–26153
nsp9	N4118-Q4230	113	12616–12954	6	63	27074–27265
nsp10	A4231-Q4369	139	12955–13371	7a	122	27273–27641
nsp11	S4370-V4382	13	13372–13410	7b	44	27638–27772
nsp12(Pol)	S4370-Q5301	932	13372–16166	8a	39	27779–27898
nsp13(Hel)	A5302-Q5902	601	16167–17969	8b	84	27864–28118
nsp14(ExoN)	A5903-Q6429	527	17970–19550	9b	98	28130–28426
nsp15(XendoU)	S6430-Q6775	346	19551–20588	14	70	28583–28795
nsp16(2′-O-MT)	A6776-N7073	298	20589–21482			

Products of ORFs 1a and 1ab with predicted processing positions are listed as non-structural proteins nsp1 through nsp16. Nucleotide positions used to clone the individual SARS-CoV ORFs and the expected amino acid length are also given. Pol: RNA polymerase; Hel: C/H,NTPase,dNTPase, 5′-to3′RNA helicase and DNA helicase, RNA 5′triphosphatase; ExoN: 3′-to-5′ exoribo-nuclease, C/H; XendoU: Uridylate-specific enodribonuclease; 2′-O-MT: 2′-O-ribose methyltransferase. Nomenclature by Snijder et al. 2003 and Thiel et al. 2003.

### Y2H screen of the individual SARS-CoV ORFs and confirmation by CoIP

**Figure 1:**
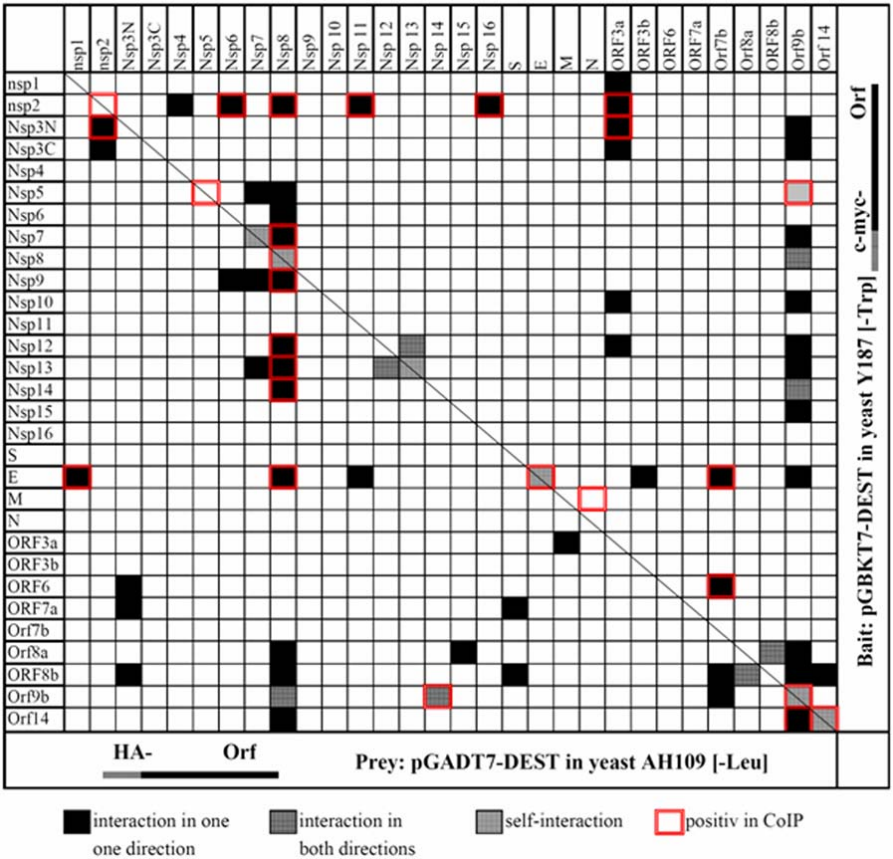
Analysis of SARS-Co viral protein interactions by Y2H matrix screen and CoIP in mammalian cells. Y2H matrix screen was performed by mating *S. cerevisiae* strains AH109 and Y187 containing prey and bait vectors with the respective SARS-CoV ORFs on selective media. All ORFs were tested against each other. Positive interactions in yeast (black and grey squares) were retested by CoIP in mammalian cells (293cells) using anti- HA (preys) and anti-c-myc (baits) antibodies. Interactions tested positive in 293 cells by CoIP are encircled in red.

The Y2H bait and prey vectors pGADT7-DEST and pGBKT7-DEST containing the SARS ORFs were transformed into the haploid yeast strains AH109 and Y187, respectively, and mated and grown under selective conditions on media lacking leucine, tryptophane and histidine. All ORFs were tested pairwise against each other and 900 individual interactions were tested in quadruplicates. Interaction of the viral proteins was indicated by colony growth on the selective plates. The result of the matrix screen is shown in Figure 1. Positive interactions are indicated by (black and patterned squares). Positive Y2H interactions were validated by co-immunoprecipitation (CoIP) in mammalian 293 cells as a second interaction test (double-lined red squares). Of 65 interactions detected by Y2H 25 were corroborated by CoIP. Four CoIPs were detected in both directions: non-structural proteins nsp12 (RdRNAP) and nsp13 (C/H, NTPAse, dNTPAse, 5′- to 3′ RNA helicase, DNA Helicase, RNA 5′- triphosphatase), nsp8 and accessory protein ORF9b, nsp14 (C/H, 3′- to 5′ exoribonuclease) and ORF9b, and accessory proteins ORF8a and ORF8b. Six of the proteins, including nsp7, nsp8, nsp13, “E”, ORF9b and ORF14 interacted with themselves indicating the formation of dimeric or multimeric complexes. Four of the self-interactions including nsp8, “E”, ORF9b and ORF14 self-interactions were also found in the CoIP assay. Two non-structural proteins nsp2 and nsp8, and the accessory protein ORF9b showed a rather large number of interactions (8, 14 and 15 interactions, respectively).

### Interactions of non-structural proteins

**Figure 2:**
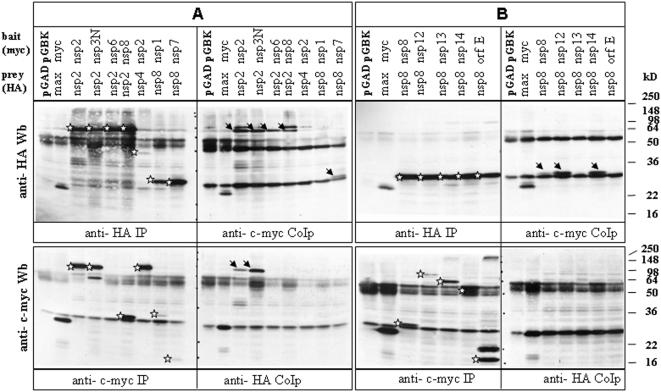
CoIPs of non-structural proteins nsp2 and nsp8. 293 cells were infected with vaccinia virus vTF-7 and subsequently co-transfected with HA- and c-myc- tagged plasmids carrying the respective SARS-CoV ORFs. After 20 hours half of the cell lysate was immunoprecipitated with anti-HA, the other half with anti- c-myc antibody. Bound proteins were subjected twice to 12,5% SDS-PAGE and Western Blot transfer, and probed cross-wise with the two antibodies. Co-precipitated proteins are indicated in the right panels. HA and c-myc tags are expressed as N-terminal fusions with the corresponding SARS-CoV ORF in plasmids pGADT7 and pGBKT7, respectively. Stars indicate expression products by IP, arrows by CoIP.

Six interactions of the non-structural proteins nsp2 could be confirmed by CoIP including the non-structural proteins nsp3N, nsp6, nsp8, nsp11 and nsp16 and ORF 3a, which only recently had been described to be a novel structural protein [22], [23]. Figure 2A shows IP and CoIP results with anti-HA and anti-c-myc antibodies: 293 cells were transfected with HA-tagged nsp2 and c-myc-tagged nsp2-, nsp3N-, nsp6- or nsp8. The lysates were split into two and immunoprecipitated in the presence of protein G with the anti- HA (left upper panel) or with the anti- c-myc (right upper panel) antibody. The bound proteins were separated by 12.5% SDS-PAGE Western blot analysis. Expressed HA-tagged proteins are indicated by stars and coprecipitated proteins by arrows. Although the nature of the additional protein species with higher molecular weight in the anti-HA WB of the anti-c-myc CoIP is unclear, they might reflect a multimeric nsp2 band that is not present in the other lanes, further supporting the nsp2-nsp2 interaction. But they could also be a result of post-translational modification upon binding to other proteins. In the reciprocal analysis (lower left and right panel) nsp2 proteins coprecipitated with itself and with nsp3N.

The second-most connected protein of the replicase complex is nsp8 (figure 2A, 2B). Of the 14 interacting proteins (nsp2, nsp5, nsp6, nsp7, nsp8, nsp9, nsp12, nsp13, nsp14, E protein, ORF8, ORF8a, ORF9b, ORF14) found in the Y2H system eight could be confirmed by CoIP (nsp2, nsp7, nsp8, nsp9, nsp12, nsp13, nsp14, E protein). Nsp 8 and nsp12 proteins interacted with ORF9b and nsp13, respectively, in both directions in the Y2H. This was also the case for the interaction between nsp14 and ORF9b, which could also be confirmed by CoIP.

### Interactions of structural proteins

Of the “classical” structural proteins S, E, M and N, only the E protein showed a number of interactions with the non-structural proteins nsp1, nsp8, nsp11, as well as with the accessory proteins ORF3b, ORF7b and ORF9b. Interestingly, M and S reacted with the only recently described accessory structural proteins ORF 3a and ORF7a [22], [24]. Self-interaction of the E protein was seen in both assays. The interaction between the structural proteins N and M, which has been described in two recent reports [21], [25], was found positive by CoIP only.

### Interactions of accessory proteins

The most dominant interactor of the accessory proteins was the ORF9b. Of the 16 ORF9b Y2H interactions detected, four could be confirmed by CoIP (Figure S1). 10 of the non-structural and five of the other accessory proteins showed interactions with other proteins. Self interactions were seen for ORF9b and ORF14. These could be confirmed by CoIP in both directions as well as the reactivity of ORF7b with the two E and ORF 6 proteins. ORF 8a and ORF 8b were also reactive in both directions in the Y2H system.

### Deletion of ORF9b from SARS-CoV

**Figure 3:**
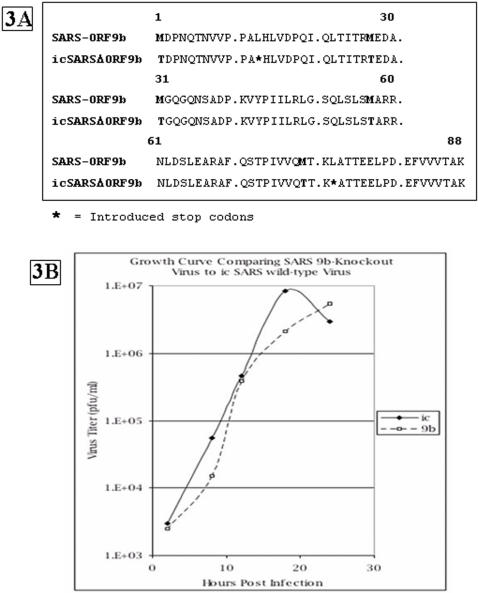
Deletion of ORF9b from SARS-CoV. DNA sequences of wild-type and of modified ATG start codons of ORF9b are given on the left. The primary sequence of the N protein was maintained. Viral growth curves are shown on the right.

The dominant interactor proteins nsp2 [17] and nsp8 (Deming et al., submitted) are dispensable or essential for viral replication *in vitro*, respectively. To determine the importance of the dominant interactor ORF9b accessory protein for viral growth a deletion mutant was made by synthesizing a DNA fragment that included changes that ablate each of the ORF9b ATG start sites while maintaining the primary sequence of the N protein (Figure 3A). The recombinant virus had normal plaque sizes and grew like wildtype virus in Vero cells after infection at a MOI of 0.1 PFU/cell (Figure 3B). Thus, deletion of ORF9b is not lethal.

### Bioinformatical analysis Comparison of SARS-CoV and herpesvirus intraviral protein interaction screens

Y2H matrix and CoIP interaction screens of the present study were performed similarly as in our recently published study on herpesviral protein networks describing intra-viral protein interactions in Kaposi sarcoma-associated herpesvirus (KSHV) and Varicella-Zoster virus (VZV) [26]. Table 2 shows the comparison of network parameters of SARS to KSHV as well as other cellular protein interaction networks. The average degree and characteristic path length are slightly smaller than in the KSHV network and significantly smaller than in the cellular networks due to smaller network size. The fraction of pairwise interactions confirmed among those tested is higher in the SARS network with 65 out of ∼450 possible non-redundant pairwise interactions ( = 14.4%) than in the KSHV network with 123 out of ∼4050 possible interactions ( = 3%). Furthermore, the clustering coefficient is significantly higher than in all of the networks analyzed. When comparing against random networks of the same size, we found that the clustering coefficient is approximately as high as expected at random given the degree distribution. Interestingly for the KSHV network it is actually smaller, whereas for the cellular networks it is much higher.

**Table 2:** Comparison of network parameters of viral and cellular protein interaction networks

Network parameters	SARS-CoV Y2H	*KSHV*	*S. cerevisiae (DIP)*	*H. sapiens*
**No.Nodes**	31	50	4959	10470
**No. Edges (including self-interactions)**	65	123	17511	45104
**Average Degree**	4.19	4.92	7.06	8.62
**Characteristic path length**	2.43	2.84	4.15	4.29
**Diameter**	5	7	11	14
**Clustering coefficient**	0.41	0.146	0.124	0.143
**Enrichment over ER**	2.92	1.56	87.12	174.3
**Enrichment over ES**	1.00	0.75	7.02	12.44

The table shows network parameters for two viral and two cellular protein interaction networks. The SARS network contains 65 interactions between 31 nodes with 6 of those interactions being self-interactions. For comparison purposes, network parameters are also shown for KSHV [26], *S. cerevisiae* and *H. sapiens*. Interactions for the cellular networks were derived from the following sources: *S. cerevisiae* from DIP (the Database of Interacting Proteins) [45], the yeast two-hybrid interactions of *H. sapiens* from the studies of Stelzl et al. [28] and literature interactions from Rual et al.[27], predicted human interactions (core) from Lehner and Fraser [29] and interactions taken from the HPRD (human protein reference database) [46]. Parameters shown include the number of nodes and edges in the networks, the average degree, characteristic path length (average shortest path), diameter (maximum shortest path), clustering coefficient and for the clustering coefficient enrichment values compared to appropriate random networks (ER and ES). Both types of random networks contain the same number of nodes and edges as the original network. ER networks [47] are created by connecting edges randomly, whereas ES networks are created by an edge swapping strategy which preserves the degree distribution (see [26], [48]). Enrichment values are calculated over the theoretical clustering coefficients of ER networks and the average clustering coefficient of 1000 randomized ES networks.

### SARS-CoV-host interaction network

**Figure 4:**
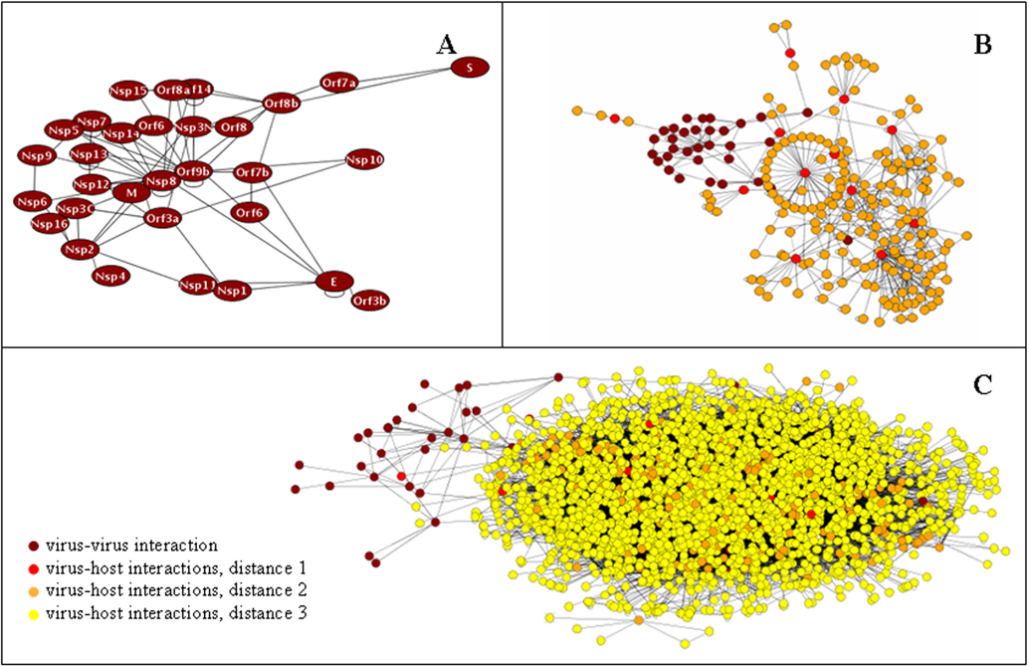
SARS-CoV-host interaction network. Viral interactions are based on the experimental Y2H findings. Human interactions were taken from the combined human interaction network described in Table 2. Interactions between SARS and human proteins were gathered from the literature and are listed in Supplemental Table 1. The figure shows the SARS interaction network (A) and proteins and interactions which are separated from the SARS network by no more than 2 (B) and 3 (C) interactions, respectively. SARS-CoV proteins are depicted in dark red, their direct targets (distance 1) in light red, neighbours of the direct targets (distance 2) in orange and neighbours of the latter (distance 3) in yellow.

Known virus-host and intraviral interactions of SARS proteins were identified by a literature screen (see Tables S1 and S2). Based on ten known SARS-host interactions as well as two interactions predicted from homologous proteins, the viral network was connected to the human network assembled from large-scale Y2H screens [27], [28], ortholog predictions [29] and literature mining ((Ref-HPRD) and [27]). The SARS-CoV-host interaction network is shown in Figure 4. Human proteins are included which are distant from the SARS-CoV proteins by at most two or three interactions as well as the interactions between these proteins. On the basis of the small number of known virus-host interactions the intraviral SARS-CoV network is separated from the bulk of the human interactome. Proteins targeted by SARS either directly or via other proteins are involved in various molecular functions and pathways such as apoptosis, cell communication and signalling pathways. The literature screen for intraviral SARS interactions identified 23 interactions. 3 of these (13%) were also determined by the Y2H screen.

### Immunofluorescence localization of SARS-CoV ORFs

To systematically study the subcellular localization of viral proteins within eukaryotic HeLa cells the SARS-CoV ORFs were transfected in eukaryotic vectors with either N- or C- terminal Flag tags and detected with an anti-Flag antibody. Since artificial tagging of proteins often leads to an aberrant expression in cellular compartments, we tagged the SARS-CoV protein on both the N- and C- terminus and only considered the cellular localization correct if consistent with both tags [30]. Some of the proteins were not expressed if tagged either N- or C- terminally (see Figure S2), and some were not expressed at all, and were not included in the analysis.

**Figure 5:**
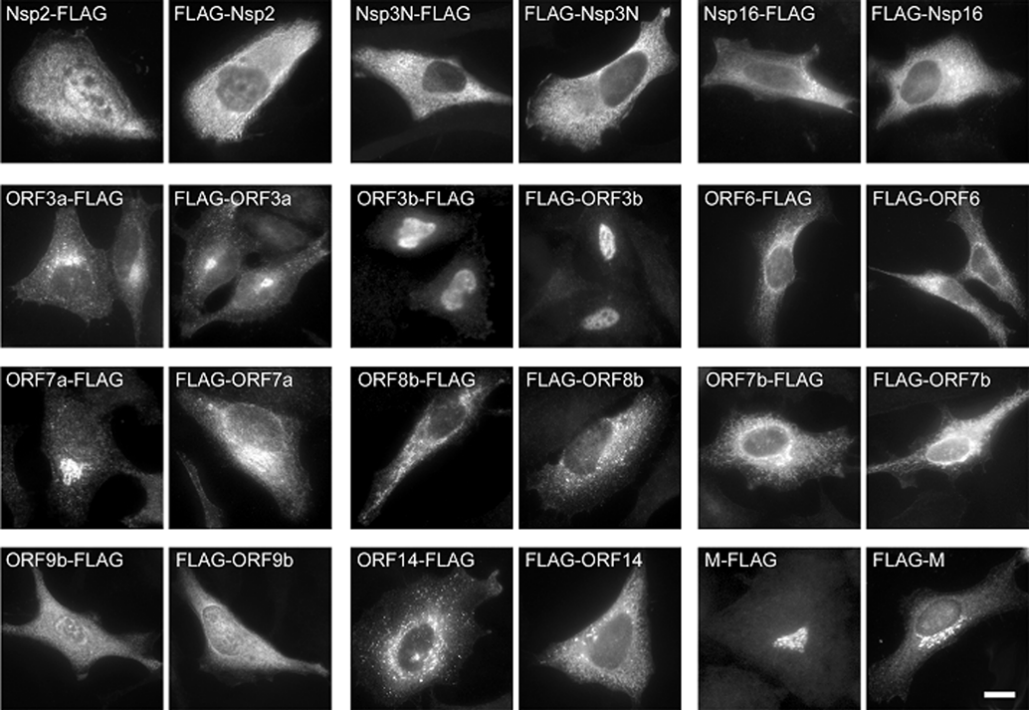
Subcellular localization analysis of SARS-CoV ORFs. Expression plasmids containing N- and C- terminally FLAG -tagged ORFs were transfected into Hela cells and analysed after 24 hours with an anti- FLAG antibody for expression and localization of their products.

ORF3a, ORF7a and M were detected in the Golgi, ORF3b in the nucleus, ORF6, ORF7b, nsp3N and nsp16 in the ER (Figure 5). ORF8b and ORF14 showed a vesicular staining, and nsp2 was found both in cytoplasm as well as in the nucleus. Orf3a and M were found to be interacting by Y2H, and were both detected in the Golgi. Similarly, ORF6 and ORF7b were both found in the ER.

## Discussion

In this study we report the cloning of the complete ORFeome of SARS-CoV and the results of a matrix-based yeast two-hybrid screen of pairwise viral protein-protein interactions. From a number of recent structural studies it is clear that during the viral life cycle large replication complexes are formed, which involve a large number of viral proteins [31]. SARS-CoV is a representative of the Coronaviridae, the largest RNA viruses known (27 to 32 kb, plus-stranded). SARS-CoV expresses at least 16 non-structural replicase proteins which are cleaved co- and post-translationally from two precursor polyproteins by two viral proteinases, four structural proteins and a set of eight accessory proteins specific for the individual virus groups [6]. Since the polypeptide processing sites of non-structural proteins are well defined, we chose the strategy to subclone all individual ORFs predicted, and not to use the precursor polyproteins. Using this approach we expected to avoid problems in the expression, folding or targeting of the polypeptides due to incorrect processing. Such problems had been reported for yeast two-hybrid assays performed with the plus-stranded RNA viruses Hepatitis C virus [32] and wheat streak mosaic virus (WSMV) [33], where interactions had been observed only when random fragments, not mature proteins were used. But there are also reports on potato virus A (PVA) and pea seed-borne mosaic virus (PsbMV), which belong to the potyvirus (+) strand RNA virus family similar to WSMV [34], as well as on a subset of poliovirus proteins [35], where interactions have been detected among cloned mature proteins.

In our screen approximately 14% of the 450 possible non-redundant protein interactions tested were positive and approximately 38% of which were confirmed by CoIP. This result is in the same order of magnitude as the outcome of similar Y2H matrix screens in KSHV and VZV, indicating that this approach can also be applied for plus-strand RNA viruses. The low numbers of Y2H interactions detected in two directions are a common phenomenon in Y2H assays and are probably due to steric constraints of either bait or prey fusion proteins.

Coronavirus replication complexes consist of intricate macromolecular structures in which many of the non-structural replicase proteins are involved. One of the most interesting interactor proteins found in our study is nsp8, which interacts with replicase proteins nsp2, nsp5, nsp6, nsp7, nsp8, nsp9, nsp12, nsp13, nsp14. The importance of this protein is supported by recently reported crystallization studies, which described the multimeric association of various of the non-strucutural proteins. Nsp 8 seems to be one of the proteins involved in these complexes. Nsp8 deletion or irreversible fusion to nsp7 or nsp 9 by mutagenesis of the corresponding cleavage site results in a lethal phenotype supporting the idea that nsp8 is absolutely essential for virus replication (Deming et al., submitted). Evidence has been presented for interaction with nsp9, a ssRNA-binding protein, by analytical ultracentrifugation experiments and by a decrease of the disorder of the nsp8 N-terminal region after the addition of nsp9 [36]. Furthermore, a hexadecameric nsp7-nsp8 supercomplex was described which was suggested to encircle RNA where it may serve as a general processivity factor for the RNA-dependent RNA Polymerase (RdRp) nsp12 (19). A very recent report described nsp8 as a second RdRp of SARS-CoV. It was shown to initiate the synthesis of complementary oligonucleotides of <6 residues in a low fidelity reaction which eventually might serve as primers for the primer-dependent nsp12 RdRp [15]. For MHV it was shown that RdRp co-immunoprecipitates with nsp8, nsp9, nsp5 and the helicase nsp13 [37], and that it also colocalizes with nsp7, nsp9 and nsp10 [18], [37]. Thus, the nsp8 interactions found by us are confirmed by a number of different studies and it seems to play an important role in the viral replication complex.

In this manuscript, we demonstrate interactions between RdRp (nsp12) and nsp8, and with the helicase nsp13 in both directions of the Y2H screen. It is likely that the RdRp interacts with more nsps than were found, but these interactions may require mediator proteins like nsp8.

Nsp2 interacted with seven other nsps including nsp8 and with one of the newly described structural proteins ORF3a. As shown by CoIP, it also self interacts to a dimeric or multimeric complex. The relatively large number of interactions might imply a crucial role of nsp2 in the viral life cycle. However, it was shown by deletion mutants of SARS-CoV and MHV that neither the encoding genomic RNA sequences nor the nsp2 proteins are necessary for the generation of infectious viruses in cell culture [17]. Since these viruses displayed slightly reduced phenotypes in growth, RNA synthesis but not protein processing, it was speculated that nsp2 might play a role in global RNA synthesis, and possibly in virus-cell interactions or viral pathogenesis. The reported subcellular localization of individually expressed nsp2 in delayed brain tumor (DBT) cells [17] is similar to the diffuse cytoplasmic and nuclear immunofluorescence staining pattern found with our N- or C- terminally tagged nsp2 proteins. Thus, the exogenously expressed nsp2 does not target specific membranes in the absence of infection. However, after coinfection with a MHV mutant virus lacking nsp2, the protein expressed *in trans* was reported to be recruited into distinct viral replication complexes. This relocalization of nsp2 to small vesicular foci in the cytoplasm was also confirmed in SARS-CoV-infected Vero cells by immunofluorescence staining with anti-nsp2 antibodies [19].

In our study, only few interactions were found for the structural proteins, which might be biased by transmembrane sequences preventing the transfer of expressed prey (containing the GAL4 activating domain) and/or bait (containing the GAL4 DNA-binding domain) fusion proteins to the nucleus of the yeast cell where protein-protein interaction leads to transcription. Only the E and ORF3a proteins showed a number of associations whose relevance is unclear. Interactions of ORF3a –M and ORF7a-S fit to the recent finding that the two accessory proteins display structural functions as has been described [24], [38].

For the group-specific accessory proteins it has recently been shown that deletion of five of the eight ORFs (ORFs 3a, ORF3b, ORF6, ORF7a and ORF7b) alone or in combination did not influence dramatically the level of RNA or the replication efficiency *in vitro* or in an *in vivo* mouse model [16]. The most interesting accessory protein with respect to interactions in our study turned out to be ORF9b. Y2H interactions with nsp8 and nsp14 were found bi-directionally and the self-interaction could also be confirmed by CoIP. Latter result is confirmed by recent structure data [39]. The ORF9b protein, which is encoded within the nucleocapsid gene, is an intertwined dimer with an amphipathic outer surface and a long hydrophobic lipid binding tunnel. This suggests that ORF9b is targeted to ER-Golgi compartments via an unusual anchoring mechanism and acts as an accessory protein during virion assembly. Although most of the accessory proteins do not seem to play pivotal roles in viral replication, they might still be important for the virus-host interplay and for pathogenicity. Currently, there is no reasonable explanation for the large number of interactions found for ORF9b by the Y2H screen. As deletion of ORF9b does not seriously reduce virus replication *in vitro* consistent with a luxury function, the 9b protein may function to enhance the global stability of the SARS proteome network and play some unrealized role in regulating virus-host protein-protein interactions. It thus might be more important for enhancing *in vivo* virulence.

Immunofluorescence localization of Flag-tagged viral proteins corresponded in most cases to published data on SARS-CoV and other coronaviruses. We found nsp2 proteins in the cytoplasm and to some extent in the nucleus which is in accordance with anti-nsp2 antibody stainings of stably DBT-nsp2 (MHV) expressing cells [17]. Many of the non-structural proteins are involved in the replication of the virus and locate to virus-induced cytoplasmic double-membrane vesicular complexes as the sites of viral replication. It is therefore important to take into account that the localization patterns of nsps might be quite different when expressed individually in cells as compared to the situation of viral infection where various viral proteins might help to recruit each other to the sites of active replication.

Accessory protein 3a, for which a number of effects on cellular functions were described [40], we located in our Flag-tagged versions to the Golgi complex as Yuan et al. [41] observed using EGFP-tagged constructs. As a structural protein ORF3a interacts with the M protein [23] which was also clearly found in the Golgi as Flag fusion proteins. The nuclear localization of ORF3b is also reasonable because it induces cell cycle arrest at the G0/G1 phase and apoptosis [42]. Proteins ORF6 and ORF7b, interacting in Y2H and CoIP, were both found in the ER. To our knowledge this localization has not been described for ORF7b before. Not much is known about ORF9b other than it is expressed in infected cells [43] and that antibodies to it are found in infected patients [44]. As opposed to Meier et al. [39], who located ORF9b to intracellular vesicular structures (293T cells), we found it to be diffusely distributed within cytoplasm and nucleus (HeLa cells).

Analysis of network statistics showed that despite high clustering coefficients the SARS interaction network is not higher clustered than expected at random. It, thus, appears as a single module such as the KSHV network and is not subdivided into separate functional modules as cellular networks. Based on currently known and predicted host-virus interactions, a joint virus-human network was derived in which the viral part of the network appears to be separated from the main host network. In this respect, the SARS network differs from the KSHV viral network which is incorporated into the host interactome. However, this may be due to the small number of virus-host interactions identified so far for SARS. Indeed for KSHV, the predicted virus-host network was based on about twice as many interactions to the host. To better understand the role of the intraviral protein interactions it is necessary to gain more knowledge on the SARS-CoV with it's host during infection.

We certainly missed a considerable number of intraviral protein interactions in our Y2H screen as can be seen for M-N and nsp2-nsp2, nsp5-nsp5 self-interactions, which we could only detect by CoIP. Although, it is generally acknowledged and certainly has to be taken into account that Y2H assays are error-prone by producing false positives and false negative results, we identified a large number of interactions which have not been reported previously and which could be confirmed biochemically. These interactions will be of great help for further studies which are aiming at the elucidation of SARS-CoV replication and pathogenesis. Future experiments with the mutant viruses lacking nsp2, nsp8 or ORF9b will show the relevance of the interactions detected for virus replication, growth and pathogenicity *in vitro* and *in vivo* model systems.

## Materials and Methods

### Experimental Procedures Viral nucleic acids

SARS-CoV orfs were derived from subcloned cDNAs described by Yount et al. [16] and Thiel et al.[7]. ORFs were amplified by nested polymerase chain reaction (PCR) using plasmids pTOPO XL containing fragments A (nt 1–4436) and B (nt4344–8712), pSMART containing fragments C (8695–12070), D (12055–18924), E (18907–24051) and F (24030–29736) as well as plasmids pMal-ScoV-Mpro 5 (nsp5), pMal-ScoV-nsp8, pET-ScoV-POL 8 (nsp12), pMal-SHEL-nsp13, pET-ScoV-ExoN 3 (nsp14), pMal-ScoV-nsp15-7, pET-ScoV-MTR 12 (nsp16)and pBS-SARSCoV-S30.

### Strategy for Recombinatorial Cloning of the SARS-CoV ORFs

The nucleotide sequences of SARS-CoV Urbani (Genbank Accession AY278741), Frankfurt (Genbank Accession AY291315) and TOR2 (Genbank Accession NC_004718) isolates were used to design primers for subcloning of all putative ORFs and making them compatible to Gateway® recombinatorial cloning system (Invitrogen). Nested PCRs were performed with two separate sets of primers. For the first PCR internal forward (AAAAAGCAGGCTCCGCCATGN_14–27_ ) and reverse (AGAAAGCTGGGTCn_13–20_ ) primers containing the internal attB1 and attB2 recombination sites were used. The gene-specific 5′ forward sequence (N) introduced an AUG start codon prior to the predicted protease cleavage sites with further 14–27 nucleotides downstream, while the 3′ reverse sequence (n) matched 13–20 nucleotides. There was no stop codon introduced at the specific ends of the predicted cleavage sites in order to allow the C- terminal inframe fusion of tag sequences. The second PCR was performed using forward (5′-GGGGACAAGTTTGTACAAAAAAGCAGGCT-3′) and reverse (5′-GGGGACCACTTTGTACAAGAAAGCTGGGT-3′) primers which included the external parts of the attB1 and attB2 recombination sites. For the putative peptide nsp11 two oligos were synthesized as the coding (5′-AAAAAGCAGGCTCCGCCATGTCTGCGGATGCATCAACGTTTTTAAACGGGTTTGCGGTGGACCCAGCTTTCT-3′) and non-coding (5′-AGAAAGCTGGGTCCACCGCAAACCCGTTTAAAAACGTTGATGCATCCGCAGACATGGCGGAGCCTGCTTTTT-3′) strands. Primers for ORFs N and S were designed such that the complete attB1 and attB2 sites were added to the gene specific sequences.

PCR conditions were 20 µM of dNTPs, 0,2–0,4 µM forward and revers primers, 20 ng of template and 1 U of Long Expand Taq Polymerase Enzyme (Roche Diagnostics GmbH). Depending on size and nucleotide composition of the amplificates two standard conditions were used for amplification. Protocol 1: 94°C for 5 min/30 cycles: 94°C for 90 sec, 52°C for 90 sec, 68°C for 210 sec/72°C for 7 min/4°C ∞. Protocol 2 (touch down): 94°C for 4 min/10 cycles: 94°C for 30 sec, 55°C (lowered by 1 degree per cycle) for 30 sec, 72°C for 90 sec/30 cycles: 94°C for 30 sec, 50°C for 30 sec, 72°C for 90 sec/72°C for 7 min/4°C ∞.

PCR fragments were separated by Agarose gel electrophoresis and purified utilizing Nucleospin Extraction Kits (Macherey& Nagel). The resulting PCR-fragments, flanked by complete attB1 and attB2 sites, were cloned by Gateway® recombinatorial cloning into the entry vectors pDONR207 or pDONR221 (Invitrogen) via the BP Clonase reaction as described by the manufacturer (Invitrogen). The overlaps of the vector and SARS-CoV-ORF sequences were confirmed by DNA sequencing using the Big Dye Terminator kit (Perkin Elmer) on a 377 DNA Sequencer, a 310 Genetic Analyser (both Applied Biosystems) or the Genome Lab DTCS-Quick Start Kit on the CEQ™ 8800 Sequencer (Beckman Coulter).

Two eukaryotic destination vectors were constructed allowing the inframe fusion of a HIS-Flag tag to the N-terminus and of a Flag-HIS tag to the C-terminus of a the viral ORFs. For the N-terminal tag a DNA oligo adaptor molecule was synthesized (coding strand: 5′-TTAGTCAAGCTTGAAGGAGATAGAGCCACCATGGCACACCATCACCATCACCATGACTACAAGGACGACGATGACAAGGCGATATCTTAATCTAGATGATA-3′) and sub-cloned via Hind III and XbaI restricition sites into plasmid pCR3. The C-terminal oligo adaptor molecule (coding strand: 5′-TTTATATGATATCGACTACAAGGACGACGATGA CAAGGCACACCATCACCATCACCATTAACTCGAGATTAATA-3′) was subcloned via EcoRV and XhoI into pCR3. Both plasmids were converted to destination vectors by ligating an EcoRV – EcoRV DNA fragment, containing the GATEWAY® conversion cassette reading frame B (rfB cassette) into their individual EcoRV sites. In the 5′- and 3′- tag vectors an inframe stop codon was introduced immediately after the EcoRV site or after the HIS tag sequence, respectively. The Y2H destination vectors pGBKT7-DEST (bait) and pGADT7-DEST (prey) were derived from pGBKT7 and pGADT7 (Clontech) by introducing a GATEWAY® rfB conversion cassette into the SmaI sites as described recently (23). Into these vectors the SARS-CoV ORFs were transferred from the donor plasmids via LR reaction. Clones were checked by restriction enzyme analysis using EcoRV for the HIS-/Flag tag vectors and EcoRI and BamHI (NEB) for the yeast vectors.

### Yeast-two-hybrid screen

The haploid *Saccharomyces cerevisiae* strains AH109 (MATa, trp1–901 m, leu2-3, 112, ura3-52, his3-200, gal4Δ, gal80Δ, LYS::GAL1_UAS_-GAL1_TATA_-HIS3, MEL1 GAL2_UAS_-GAL2_TATA_-ADE2, URA3::MEL1_UAS_-MEL1_TATA_-lacZ) and Y187 ( MATα, his3-200, trp1-901, ade2-101, ura3-52, leu2-3, 112, gal4Δ, met, gal80Δ, URA3::GAL1_UAS_-GAL1_TATA_-lacZ, MEL1) were chosen for the yeast-two hybrid assay. AH109 and Y187 were transformed using 1 µg of prey (pDEST-GADT7) or bait vector (pDEST-GBKT7), respectively. Yeast cells were incubated for 1 h in 750 µl PEG/Bicine solution (40% PEG 1000, 200 mM Bicine pH 8.35) at 30°C, followed by 5 min at 45°C. Cells were pelleted and resupended in 1 ml NP-buffer (0.15 M NaCl, 10 mM Bicine pH 8.35), pelleted a second time and resuspended in 200 µl NP-buffer and plated on to SD medium (+2% agar) lacking either leucine (prey) or tryptophane (bait). Colonies were visible after 2–3 days. The yeast strains AH109 and Y187 containing proteins in prey and bait were arrayed in a 96-deep-well plates with SD liquid media lacking leu or trp according to the interactions to be tested. The liquid cultures were transferred on to SD medium plates lacking leu or trp using a 384-pin replica tool (Nunc). Colonies were grown for 2 days at 30°C and used directly for mating on YPD medium plates. Each mating was performed in quadruplicates, and after 2 days at 30°C the colonies were stamped onto SD medium (–Leu–Trp) plates. The interactions were assessed by transfer to SD–Leu–Trp–His plates, and interactions considered positive if at least three out of four possible colonies grew. Viral proteins acting as self-activating baits were analyzed on increasing amounts of +3 mM 3-Amino-1,2,4-triazole (3AT, Sigma) (3 mM, 10 mM, 50 mM and 100 mM), and excluded from the results if no clear positive interactions could be determined.

### Co-immunoprecipitation

293 cells were infected with recombinant vaccinia virus vTF-7 expressing the T7 RNA polymerase (NIH AIDS repository) at a MOI of 10 in DMEM/1% FCS. One hour post infection, virus-containing medium was removed and substituted by DMEM/1% FCS. Ten micrograms (per dish) of the respective pGBKT7-SARS-CoV-ORF and pGADT7- SARS-CoV-ORF plasmids were then transfected into two 10 cm dishes of 293 cells using the calcium phosphate method. After 20 to 24 h, cells were lysed by incubation in NP-40 lysis-buffer (1% NP-40, 140 mM NaCl, 5 mM MgCl_2_, 20 mM Tris pH 7.6, 1 mM PMSF, one tablet of Complete protease inhibtor cocktail (Roche) per 50 ml on ice for 30 min. Lysates were centrifuged at 20.500× g for 10 min and precleared using 50 µl preequilibrated protein G-sepharose (Amersham Pharmacia). Lysates were precipitated using either 5 µl (200 µg/ml) mouse monoclonal anti-myc (Santa Cruz Biotechnology) or 10 µl (100 µg/ml) rat monoclonal anti-HA (Roche Diagnostics GmBH) antibodies in the presence of 50 µl protein G Sepharose beads and incubated ON at 4°C by overhead rotation. The beads were washed 3 times in ice-cold NP-40 buffer and resuspended in 2× SDS protein sample buffer. Precipitates were separated by SDS-PAGE using 12.5% or 15% polyacrylamide gels. Proteins were transferred to nitrocellulose (Schleicher & Schuell) membranes in Western blot chambers ON at 4°C. Filters were blocked with 5% milk powder in TBST (50 mM Tris, pH7.6, 150 mM NaCl, 0,05% Tween-20) for 1 h. They were then incubated with the anti-myc and anti-HA antibodies at dilutions of 1∶1000 ON at 4°C. Filters were washed three times for 10 min with TBST. Incubation with the secondary, peroxidase-conjugated anti-mouse IgG or anti-rat IgG (1∶3000 each) antibodies (Jackson) was carried out for two to three hours. After three further washing steps filters were developed using the ECL™ Western Blotting Detection Kit (Amersham Biosciences). The CoIP was scored positive if a coprecipitate was detected in at least one direction.

### Recombinant virus

Recombinant SARS-CoV technology was done as described by Yount et al. [16]. The sequence of the mutated SARS-CoV ORF9b knockout (icSARSΔORF9b) was confirmed by sequencing cDNA isolated from recombinant virus.

### Immunofluorescence

Subcellular localization analysis of ORFs was carried out in HeLa cells (ATCC CCL-2). Cells were transfected with the plasmids using FuGENE6 (Roche) according to the manufacturer's instructions, for a total of 24 h. Cells were then fixed in ice cold methanol prior to processing for immunofluorescence. Primary mouse anti-FLAG (M2) (Sigma) and anti-mouse-Alexa488-conjugated secondary antibodies (Molecular Probes) were used to detect transfected cells, and coverslips were mounted in Mowiol. Images were acquired on a Zeiss Axiovert 200 microscope with a 63×/1.4 NA oil objective and standard filter sets.

## Supporting Information

Figure S1 CoIPs of accessory proteins. 293 cells were infected with vaccinia virus vTF-7 and subsequently co-transfected with HA- and c-myc- tagged plasmids carrying the respective SARS-CoV ORFs. After 20 hours half of the cell lysates was immunoprecipitated with anti- c-myc, the other half with anti- HA antibody (left panel). Bound proteins were subjected twice to 15% SDS-PAGE and Western Blot transfer, and probed cross-wise with the two antibodies. Co-precipitated proteins are indicated in the right panel. HA and c-myc tags are are expressed as N-terminal fusions with the corresponding SARS-CoV ORF in plasmids pGADT7 and pGBKT7, respectively.
(0.20 MB TIF)

Figure S2 Subcellular localization analysis of SARS-CoV ORFs. Expression plasmids containing N- or C- terminally FLAG -tagged ORFs were transfected into Hela cells and analysed after 24 hours with an anti-Flag antibody for expression and localization of their products. For these ORFs either the N- or the C- terminally FLAG-tagged ORF was detected.
(0.21 MB TIF)

Table S1 Virus-host interactions from literature screen. The table shows previously published interactions between SARS proteins and their human targets as well as two interactions which were predicted for SARS from interactions between homologous proteins. Literature interactions were determined by manually screening Medline-abstracts on SARS and related coronaviruses.
(0.03 MB DOC)

Table S2 Virus-virus interactions from literature screen. The table shows previously published interactions among SARS proteins. Interactions which were confirmed by our yeast two-hybrid screen are marked in red. As for the virus-host interactions, literature interactions were determined by manually screening Medline-abstracts on SARS and related coronaviruses.
(0.04 MB DOC)
