# Supplementary figures and images for: Analysis of Intraviral Protein-Protein Interactions of the SARS Coronavirus ORFeome
Source: PLoS One. 2007 May 23;2(5):e459. doi: 10.1371/journal.pone.0000459 (PMC1868897; doi:10.1371/journal.pone.0000459)

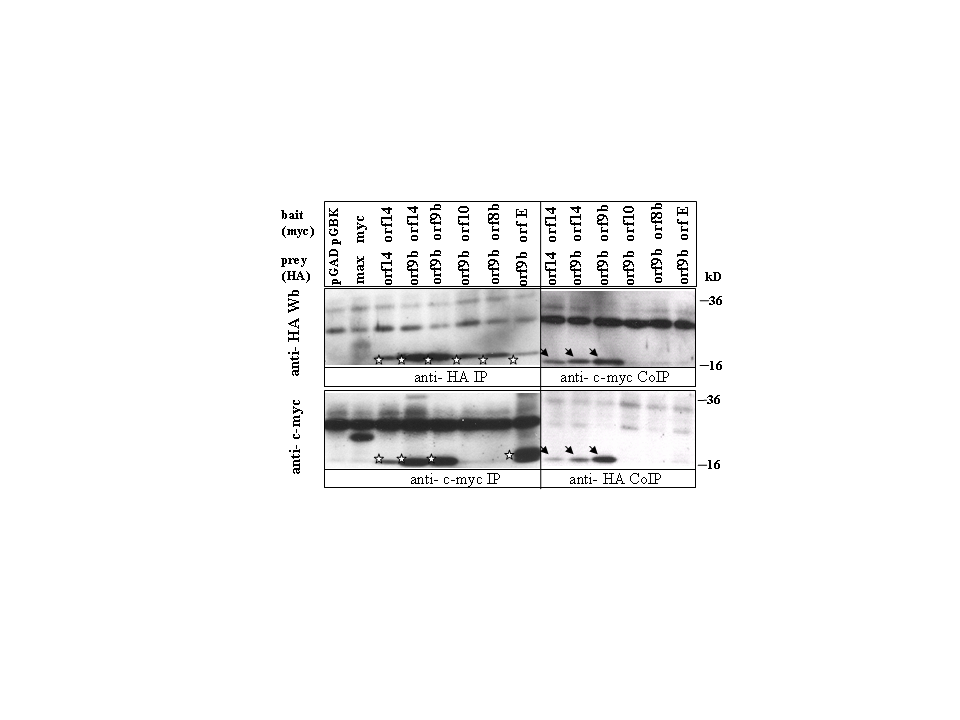

Supplement: Figure S1 — CoIPs of accessory proteins. 293 cells were infected with vaccinia virus vTF-7 and subsequently co-transfected with HA- and c-myc- tagged plasmids carrying the respective SARS-CoV ORFs. After 20 hours half of the cell lysates was immunoprecipitated with anti- c-myc, the other half with anti- HA antibody (left panel). Bound proteins were subjected twice to 15% SDS-PAGE and Western Blot transfer, and probed cross-wise with the two antibodies. Co-precipitated proteins are indicated in the right panel. HA and c-myc tags are are expressed as N-terminal fusions with the corresponding SARS-CoV ORF in plasmids pGADT7 and pGBKT7, respectively. (0.20 MB TIF) [file pone.0000459.s001.tif]

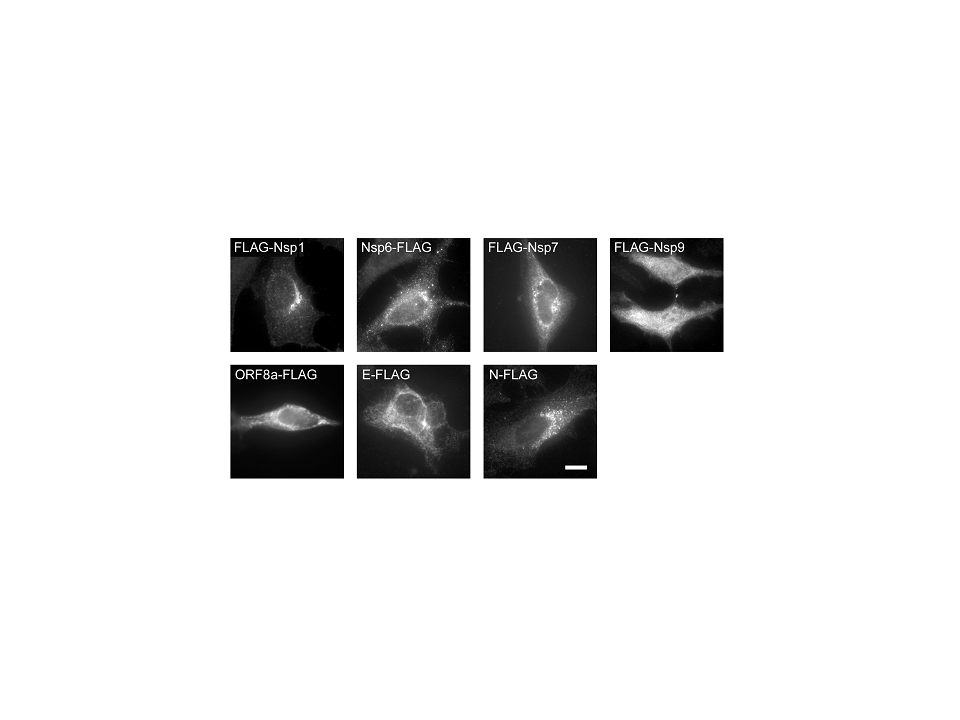

Supplement: Figure S2 — Subcellular localization analysis of SARS-CoV ORFs. Expression plasmids containing N- or C- terminally FLAG -tagged ORFs were transfected into Hela cells and analysed after 24 hours with an anti-Flag antibody for expression and localization of their products. For these ORFs either the N- or the C- terminally FLAG-tagged ORF was detected. (0.21 MB TIF) [file pone.0000459.s002.tif]
