# Supplementary material for: Analysis of Intraviral Protein-Protein Interactions of the SARS Coronavirus ORFeome
Source: PLoS One. 2007 May 23;2(5):e459. doi: 10.1371/journal.pone.0000459 (PMC1868897; doi:10.1371/journal.pone.0000459)
Supplement: Table S1 — Virus-host interactions from literature screen. The table shows previously published interactions between SARS proteins and their human targets as well as two interactions which were predicted for SARS from interactions between homologous proteins. Literature interactions were determined by manually screening Medline-abstracts on SARS and related coronaviruses. (0.03 MB DOC) [file pone.0000459.s003.doc]

| **SARS** | **Human** | **Symbol** | **Gene-Id** | **PMID** |
| --- | --- | --- | --- | --- |
| **Known** | | | | |
| S | ACE2 | ACE2 | 59272 | 14647384 |
| E | bcl-xL | BCL2L1 | 598 | 16048439 |
| N | hnRNP-A1 | HNRPA1 | 3178 | 15862300 |
| ORF7a | hSGT | SGTA | 6449 | 16580632 |
| Nsp5 | V-ATPase G1 subunit | ATP6V1G1 | 9550 | 16226257 |
| Nsp10 | BTF3 | BTF3 | 689 | 16157265 |
| Nsp10 | ATF5 | ATF5 | 22809 | 16157265 |
| Nsp10 | NADH 4L | ND4L | 4539 | 16157265 |
| Nsp10 | cytochrome oxidase II. | COX2 | 4513 | 16157265 |
| N | cyclophilin A (CyPA) | PPIA | 5478 | 15688292 |
|  |  |  |  |  |
| **Predicted from interactions of homologous proteins** | | | | |
| S | mCEACAM1a | CEACAM1 | 634 | 15749126 |
|  | nucleolin | NCL | 4691 | 11967337 |
