# Supplementary material for: Analysis of Intraviral Protein-Protein Interactions of the SARS Coronavirus ORFeome
Source: PLoS One. 2007 May 23;2(5):e459. doi: 10.1371/journal.pone.0000459 (PMC1868897; doi:10.1371/journal.pone.0000459)
Supplement: Table S2 — Virus-virus interactions from literature screen. The table shows previously published interactions among SARS proteins. Interactions which were confirmed by our yeast two-hybrid screen are marked in red. As for the virus-host interactions, literature interactions were determined by manually screening Medline-abstracts on SARS and related coronaviruses. (0.04 MB DOC) [file pone.0000459.s004.doc]

| **Protein 1** | **Protein 2** | **PMID** | **Y2H** |
| --- | --- | --- | --- |
| E | E | 16698774 | 1 |
| M | N | 15351485 | 0 |
| N | N | 16627473 | 0 |
| Nsp15 | Nsp15 | 16882730 | 0 |
| Nsp5 (3CLpro) | Nsp5 (3CLpro) | 16329994 | 0 |
| Nsp9 | Nsp9 | 14962394 | 0 |
| Orf3a | E | 15194747 | 0 |
| Orf3a | M | 15194747 | 1 |
| Orf3a | Orf3a | 16894145 | 0 |
| Orf3a | S | 15194747 | 0 |
| Orf7a | E | 16580632 | 0 |
| Orf7a | M | 16580632 | 0 |
| Orf7a | Orf3a | 15194747 | 0 |
| Orf7a | S | 16840309 | 1 |
| Orf8a | S | 16876844 | 0 |
| Orf8ab | Orf3a | 16876844 | 0 |
| Orf8ab | Orf7a | 16876844 | 0 |
| Orf8ab | S | 16876844 | 0 |
| Orf8b | E | 16876844 | 0 |
| Orf8b | M | 16876844 | 0 |
| Orf8b | Orf3a | 16876844 | 0 |
| Orf8b | Orf7a | 16876844 | 0 |
| S | S | 14996844 | 0 |
